# A Look at the Past History of Hepatitis E in Haiti: Should it be a Warning Sign during the Current Crisis?

**Published:** 2010-03-01

**Authors:** Seyed Moayed Alavian

**Affiliations:** 1 Baqiyatallah Research Center for Gastroenterology and Liver Disease,Baqiyatallah University of Medical Sciences, Tehran, I.R.Iran

**Keywords:** Hepatitis E, Haiti, Crisis, Earthquake, Prevention

## Abstract

Nobody can forget the devastating 7.0magnitude earthquake that struck poverty-stricken Haiti, Port-au-Prince recently on 12 January 2010. At least 75,000 people were killed and hundreds of thousands became homeless; authorities are worried about sanitation and outbreaks of disease in the region. The camps are full of people and there are not even the most basic facilities for any others. Humanity obliges us to help them in any possible way. I reviewed the literature about the hepatitis E virus infection in Haiti and I would like to draw the scientists’ attention to this important topic in this time of crisis.

## Introduction

The Hepatitis E virus (HEV), a small non-enveloped RNA virus, is a causative agent of acute hepatitis especially found in developing countries. HEV is an enterically transmitted viral disease of great importance to public health in the developing countries [1][2].HEV has a worldwide distribution, but predominating factors include tropical climates, inadequate sanitation, and poor personal hygiene. Outbreaks are associated with rainy seasons, floods when sewage water gain access to open water reservoirs [3], and overcrowding [4]. Since outbreaks occur via transmission of fecally contaminated drinking water, HEV is an especially important health hazard after heavy rain in endemic areas that have inadequate or disrupted sanitation systems. Use of river water for drinking, cooking, personal washing, and disposal of human excreta appears to be closely associated with the prevalence of infection. Outbreaks of HEV infection have been reported in wartime, and after the contamination of water supplies in the past [5]. The World Health Organization (WHO) has reported thousands of cholera cases in Iraq and Afghanistan in 2007 which demonstrate the limited access of people to safe water in those countries. The infrastructure of the water supply has been totally damaged as a result of war. There are many reports of HEV outbreaks due to catastrophic health conditions in Iraq and Afghanistan. Transmission of HEV occurs primarily by the fecal-oral route through contaminated water supplies in developing countries [5]. Young people are at high risk for acquiring the infection [6]. First, it was thought that HEV can occur only in developing countries including Africa and Asia, but there are many reports from Central Asian republics of the former Soviet Union, and also from Mexico. HEV has a worldwide distribution and unfortunately it is a neglected infection in many countries [7][8][9]. The prevalence in blood donors in every country may point to the vulnerable element of the population [10]. In earthquakes when the infrastructure of the water supply and the integrity of families are damaged significantly, the prevalence of E virus infection may increase. This event can continue until the restoration of the water supply to the families [11]. The socio-economic condition, crowded living environment and educational level of the affected families are other important factors [11]. In a study among children living in post-earthquake camps after the disaster in Duzce and Golyaka (Turkey), a higher prevalence of enterically transmitted infection was found. This study has pointed out the importance of providing for the urgent need for sufficient sanitary facilities after disasters in order to prevent or reduce the incidence of enterically transmitted hepatitis, especially in regions which were at risk for various disasters. Essential health precautions such as providing safe water and food supplies must be taken and an emergency action plan for preventing the infectious disease must be prepared before and after disasters such as earthquakes [12].

## Haiti and HEV

Travelling and increased communication between communities that are living in different countries can facilitate the transmission of different pathogens and the introduction of new pathogens into new geographic areas and exposes non-immune individuals to infection. In 1995 four cases of acute HEV infection with jaundice were identified among Bangladeshi soldiers whose battalion had recently been deployed to Haiti as peacekeepers [13]. This concerned the United Nations Mission in Haiti (UNMIH), who were conducting an epidemiological serological investigation for better understanding the threat of HEV in UNMIH and native Haitians. The findings showed the highest prevalence of HEV among those military contingents from the Indian subcontinent where HEV is known to be endemic [13]. A small proportion (3%) of Haitian civilians were positive for serological testing of HEV. These results suggest that HEV was not endemic in Haiti and that concern about its importation by multinational peacekeepers (UNMIH) with acue disease was justified. However, introduction of a pathogen such as HEV into a new geographic area is just one part of a complex process that may lead to the emergence of disease in Haiti. People who have never contracted HEV are at risk for acquiring the infection during the crisis. Surveys conducted in other parts of Central and South American countries showed the prevalence rate of past HEV infection (Anti-HEV Ab): Nicaragua (5–8%) [14], Brazil (6%) [15], Chile (7%) [16], Peru (14%) [17], and Venezuela (2–5%) [18]. There are two reports of outbreaks of HEV infection in Mexico in 1986 [19]. Similar to the survey in Nicaragua, in the study in Haiti, 5–6% of the soldiers sampled from Guatemala and Honduras had evidence of past HEV infection [13].Unfortunately, the HEV infection has been introduced to Haiti’s ecosystem and the conditions that appeared favorable to the emergence of disease in many communities, including poor sanitary conditions and contaminated water supplies, are present over there. Moreover, Haiti lies within the Caribbean’s hurricane belt. Environmental health conditions can be made even worse by fresh water flooding and mud slides after natural disasters, with heavy rainfalls such as those which occurred in southern Haiti with Hurricane Gordon in 1994 [13].The situation in Haiti is catastrophic now; the infrastructure of urban living is totally destroyed and many soldiers and rescuers have entered Haiti. The health system should be aware of the probability of an outbreak of HEV infection.

## Prevention Strategies

The only prophylactic measures against HEV infection that can be currently recommended are improved sanitation and especially sanitary handling of food and water. The starting of educational programs to stress sanitary disposal of feces and careful hand washing after defecation and before handling food is important. The availability of clean water supplies reduces the risk of contraction, and boiling the water appears to inactivate the HEV [5][19][20]. Essential precautions such as providing clean water and food supplies must be taken and an emergency action plan for preventing the infectious disease must be prepared before disasters such as earthquakes. To sum up the issues, immediate provision for basic urgent needs, the burying of dead bodies of humans and animals away from water supplies, the educating of people to avoid using unsafe and unboiled water, the reporting of acute hepatitis cases with jaundice and isolating them, and the integrating of the surveillance system for the control of hepatitis infection are essential.To do the above, there is an urgent need for international action, tomorrow may be too late!

## References

[1] Arankalle VA, Chadha MS, Tsarev SA, Emerson SU, Risbud AR, Banerjee K, Purcell RH (1994). Seroepidemiology of water-borne hepatitis in India and evidence for a third enterically-transmitted hepatitis agent. Proc Natl Acad Sci U S A.

[2] Chow WC, Lee AS, Lim GK, Cheong WK, Chong R, Tan CK, Yap CK, Oon CJ, Ng HS (1997). Acute viral hepatitisE: clinical and serologic studies in Singapore. J Clin Gastroenterol.

[3] Sreenivasan MA, Sehgal A, Prasad SR, Dhorje S (1984). A sero-epidemiologic study of a water-borne epidemic of viral hepatitis in Kolhapur City, India. J Hyg (Lond)..

[4] Ghorbani GA, Alavian SM, Esfahani AA, Assari SH (2007). Seroepidemiology of Hepatitis E Virus in Iranian Soldiers. Hepat Mon.

[5] Alavian SM, Fallahian F, Lankarani KB (2009). Epidemiology of Hepatitis E in Iran and Pakistan. Hepat Mon.

[6] Shamsizadeh A, Nikfar R, Makvandi M, Shamsizadeh N (2009). Seroprevalence of Hepatitis E Infection in Children in the Southwest of Iran. Hepat Mon.

[7] (1993). Hepatitis E among U.S. travelers, 1989-1992. MMWR Morb Mortal Wkly Rep.

[8] Alavian SM (2007). Hepatitis E Virus Infection: A Neglected Problem in Our Region. Hepat Mon.

[9] Meng XJ, Dea S, Engle RE, Friendship R, Lyoo YS, Sirinarumitr T, Urairong K, Wang D, Wong D, Yoo D, Zhang Y, Purcell RH, Emerson SU (1999). Prevalence of antibodies to the hepatitis E virus in pigs from countries where hepatitis E is common or is rare in the human population. J MedVirol.

[10] Keyvani H, Shamsi Shahrabadi M, Najafifard S, Hajibeigi B, Fallahian F, Alavian SM (2009). Seroprevalence of anti-HEV and HEV RNA among volunteer blood donors and patients with hepatitis B and C in Iran. Bangladesh Liver Journal.

[11] Kaya AD, Ozturk CE, Yavuz T, Ozaydin C, Bahcebasi T (2008). Changing patterns of hepatitis A and E sero-prevalences in children after the 1999 earthquakes in Duzce, Turkey. J Paediatr Child Health.

[12] Sencan I, Sahin I, Kaya D, Oksuz S, Yildirim M (2004). Assessment of HAV and HEV seroprevalence in children living in post-earthquake camps from Duzce, Turkey. Eur J Epidemiol.

[13] Gambel JM, Drabick JJ, Seriwatana J, Innis BL (1998). Seroprevalence of hepatitis E virus among United Nations Mission in Haiti (UNMIH) peacekeepers, 1995. Am J Trop Med Hyg.

[14] Perez OM, Morales W, Paniagua M, Strannegard O (1996). Prevalence of antibodies to hepatitis A, B, C, and E viruses in a healthy population in Leon, Nicaragua. Am J Trop Med Hyg.

[15] Pang L, Alencar FE, Cerutti C Jr, Milhous WK, Andrade AL, Oliveira R, Kanesa-Thasan N, MaCarthy PO, Hoke CH Jr (1995). hepatitis E infection in the Brazilian Amazon. Am J Trop Med Hyg.

[16] Ibarra H, Riedemann S, Reinhardt G, Frieck P, Siegel F, Toledo C, Calvo M, Froösner G (1997). [Prevalence of hepatitis E virus antibodies in blood donors and other population groups in southern Chile].. Rev Med Chil.

[17] Hyams KC, Yarbough PO, Gray S, Callahan J, Gotuzzo E, Gutierrez J, Vasquez PB, Hayes CG, Watts DM (1996). Hepatitis E virus infection in Peru. Clin Infect Dis.

[18] Pujol FH, Favorov MO, Marcano T, Esté JA, Magris M, Liprandi F, Khudyakov YE, Khudyakova NS, Fields HA (1994). Prevalence of antibodies against hepatitis E virus among urban and rural populations in Venezuela. J Med Virol.

[19] Velazquez O, Stetler HC, Avila C, Ornelas G, Alvarez C, Hadler SC, Bradley DW, Sepúlveda J (1990). Epidemic transmission of enterically transmitted non-A, non-B hepatitis in Mexico, 1986-1987. Jama.

[20] Corwin A, Jarot K, Lubis I, Nasution K, Suparmawo S, Sumardiati A, Widodo S, Nazir S, Orndorff G, Choi Y (1995). Two years’ investigation of epidemic hepatitis E virus transmission in West Kalimantan (Borneo), Indonesia. Trans R Soc Trop Med Hyg.

